# Multi-Body Dynamic Analysis of Hydrostatic Bearing with the MMC Material in Micro-Nano Machining

**DOI:** 10.3390/mi14091734

**Published:** 2023-09-04

**Authors:** Ali Khaghani, Atanas Ivanov, Kai Cheng

**Affiliations:** Department of Mechanical and Aerospace Engineering (MAE), Brunel University London, Uxbridge UB8 3PH, UK; atanas.ivanov@brunel.ac.uk (A.I.);

**Keywords:** micromachining, ultraprecision machining, hydrostatic bearing, MMC material, linear slide, frequency response, harmonic response

## Abstract

This study focuses on the analysis of a linear hydrostatic bearing using harmonic frequency response and harmonic response simulations. The aim is to evaluate the feasibility of replacing the existing alloy steel material with a metal matrix composite (MMC) in terms of its performance and dynamic characteristics for both the base and carriage parts. The simulation results indicate that the MMC material exhibits higher resonant frequencies and improved damping capabilities compared to the structural steel material. The higher resonant frequencies observed in the MMC material are attributed to its stiffness and structural properties. These properties contribute to increased natural frequencies and improved vibration damping characteristics. This suggests that incorporating the MMC material in the bearing design could enhance motion control, improving the ability to precisely control and manipulate the movement of components or systems. In the context of ultraprecision machining applications, incorporating the MMC material in the hydrostatic bearing design can also lead to a more accurate and controlled motion, resulting in improved precision and finer machining outcomes. The displacement analysis confirms that both materials meet the specifications provided by the manufacturer, supporting the viability of using MMC as an alternative. However, further experimental validation and considerations of material feasibility, manufacturing factors, and cost-effectiveness are necessary before implementing the MMC material in practical applications. Overall, this research highlights the potential benefits of MMC in the design of linear hydrostatic bearings, paving the way for enhanced performance in ultraprecision machining processes.

## 1. Introduction

Linear slideways with hydrostatic or aerostatic bearings are commonly used micromachining and ultraprecision machining systems, particularly in combination with slow tool servo (STS) and fast tool servo (FTS) techniques. However, based on a review of the literature, machining freeform surfaces with the STS technique can present challenges in terms of the dynamics of linear slide motion performance and functionality. Achieving sub-micron level motion accuracy is often challenging due to factors such as mechanical vibrations, thermal expansion, system backlash, dynamic response of control systems, and environmental disturbances. These factors introduce errors and uncertainties that hinder the achievement of precise positioning at the sub-micron level. For instance, in different application, high dynamics prevent electrode burning and optimize machining efficiency in wired EDM machines. The utilisation of frictionless slides and the implementation of lightweight materials with high stiffness contribute to improved precision and repeatability in positioning [1]. Therefore, a reliable slideway design is crucial in machine design and must be capable of eliminating external impacts such as thermal expansion, vibrations, and frictions. The load-carrying capacity in a hydrostatic bearing, typically using high-pressure liquid such as oil, is generated externally [2,3].

The high-pressure liquid used in hydrostatic bearings, typically oil, can result in a wide range of viscous damping effects. The bearing clearance between the stationary and movable carriage typically falls within the range of 10 to 100 μm. Due to the high precision engineering requirements, the design, development, and manufacturing of hydrostatic bearing components are limited to a few manufacturers. In ultraprecision machine and component design, such as diamond turning machines, hydrostatic bearings are custom-manufactured based on customer requirements, without a standard for oil pressure and resource. Therefore, the design of a hydrostatic bearing is dependent on the maximum load capacity needed for a particular application. The design of the supply pressure in hydrostatic bearings is influenced by two key factors: speed and load capacity. If a higher load capacity is required, the hydrostatic bearing must be designed with a higher supply pressure to maintain maximum loads. On the other hand, if the focus is on speed and temperature, a lower pressure would be more appropriate. As a result, the design of an ultraprecision machining (UPM) system that uses hydrostatic bearings is directly linked to the load capacity and the supply of high-pressure oil [4].

Hydrostatic bearings using incompressible liquid and high-pressure offer several advantages, including high damping, high static stiffness, proper pressure distribution in the presence of bearing surface imperfections, and high load-carrying capacity [5]. The ratio of error motion in a hydrostatic bearing is typically below 100 nm, even with imperfect bearing surface finishing [6]. However, hydrostatic bearings have limitations in their performance due to a higher dynamic friction coefficient at higher linear speeds. The dynamic friction in a linear hydrostatic bearing is typically low and can often be neglected at lower speeds. When using hydrostatic bearings in ultraprecision machining (UPM) spindles with high angular velocities, it is important to reduce power losses. The STS technique typically utilizes hydrostatic bearings due to their robust stiffness and damping in bearing load and acceptable linear accuracy in the back and forward motion steps during the machining process [7]. However, there are limitations with regard to speed and acceleration when using hydrostatic bearings in STS mode. If the velocity is too high, thermal and heat expansion in the contact surfaces of the slides will rapidly increase due to constant and repeatable friction. The oil film gap also has a correlation with thermal and heat generation in linear hydrostatic bearings, which can affect their linear motion accuracy [8].

This research work aims to quantify the linear motion accuracy of hydrostatic bearings used in ultraprecision machining (UPM) systems by evaluating their straightness or linearity. Although hydrostatic bearings have a high level of repeatability in their constant back-and-forth linear motion, investigating their motion accuracy is a priority. The main focus of this study is to explore the potential of using lightweight materials, specifically metal matrix composites (MMCs), to enhance the motion robustness and system dynamics of hydrostatic bearing slideways in ultraprecision machining techniques. The objective is to investigate the feasibility and dynamic characteristics of incorporating MMCs in order to optimize the performance and stability of the system, particularly in STS mode. This is motivated by the potential benefits of lightweight materials, including improved motion control, reduced inertia, and enhanced structural stability.

## 2. Metal Matrix Composite Material

Composite materials, also known as composites, are created by combining two or more base engineering materials to form a new material with unique properties. These composites can be classified into categories such as metal, polymer, and ceramic composites. In various industrial engineering fields, metal matrix composites (MMCs) have been extensively used for prototyping in space innovation, the automotive industry, commercial airliners, as well as in component manufacturing and sustainable design systems [9]. While many matrix composites, including those with higher material densities, offer desirable operational characteristics, aluminium-based composites are particularly noteworthy due to their excellent wear resistance and damping properties. However, many applications require matrix properties provided by superalloys such as titanium and magnesium. While these new materials offer structural benefits, their processing can be challenging, particularly when integrating materials with low wettability of reinforcement particles within the molten metal matrix [10]. This phenomenon hinders synthesis through conventional processing methods. Metal matrix composites (MMCs) offer several advantages in various applications. They exhibit corrosion and wear resistance, leading to enhanced durability and longevity. Additionally, the incorporation of reinforcing particles results in increased mechanical strength and stiffness, improving overall structural performance [11,12].

MMCs also demonstrate a decrease in fatigue, providing enhanced reliability under cyclic loading conditions. However, it is important to consider the associated disadvantages. Firstly, MMCs can be costly due to the materials involved and specialized fabrication processes required. Furthermore, the manufacturing process can be time-consuming, requiring careful attention to detail. Additionally, working with MMCs may involve exposure to chemicals and/or solvents, necessitating proper safety measures. Lastly, specialized equipment is often necessary for the fabrication and processing of MMCs. Therefore, while MMCs offer notable advantages, careful consideration of the associated disadvantages is essential in their application [13]. Nevertheless, emerging technologies, including laser powder bed fusion (LPBF) and advanced simulation models, are showing promise in addressing the inherent challenges of AL-MMC production. As AL-MMC manufacturing remains a complex task, leveraging these innovations might pave the way for more efficient and robust production techniques [14,15]. Metal matrix composites (MMCs) exhibit enhanced material properties when reinforcement particles are incorporated, improving mechanical characteristics such as yield strength, tensile strength, creep resistance, and thermal stability. Aluminium-matrix composites, similarly to other composites, consist of tailored combinations of materials to achieve specific mechanical properties. Compared to other MMCs, aluminium composites offer cost advantages along with superior thermal conductivity, non-flammability, high-temperature operation, high shear strength, excellent abrasion resistance, and minimal surface degradation when exposed to corrosives or solvents. Additionally, MMCs can be manufactured using conventional processes [9,16]. Various techniques such as powder metallurgy, physical vapor deposition, stir casting, hot isostatic pressing, electrical discharge machining (EDM), in situ reinforcement development, squeeze casting, and short or long fiber pressing methods are employed to produce aluminium MMCs. Notably, reducing the size of reinforcement particles to the nano level significantly affects the material’s mechanical properties, particularly wear behavior, by strengthening particle interactions [17,18].

The utilization of MMC materials for hydrostatic bearing components, particularly in the contact between the guide rail and the workbench slider, poses a question worth exploring. However, the low wettability of ceramic particles with the molten base metal matrix presents challenges in component fabrication, limiting the use of traditional casting processes. Modified fabrication processing methods, such as those employed in powder metallurgy, are preferred to overcome this issue. In powder metallurgy, small powder aggregates tend to form molecular to macro clusters, hindering their uniform dispersion within the matrix structure. The manufacturing of MMC materials can be categorized into two main groups: ex situ and in situ processes. Ex situ synthesis involves adding molecular reinforcement to the molten liquid or powdered metal, while in situ methods generate ceramic molecular/nano compounds through reactions during processing [19,20]. Comparing MMC materials with monolithic metals reveals several advantages of MMCs. These materials offer higher strength-to-density and stiffness-to-density ratios, improved fatigue resistance, superior elevated temperature properties, lower creep rate, lower coefficients of thermal expansion, and better wear resistance. To contribute to this research work, a numerical analysis is required to study the dynamic behavior of linear slideways when they are constructed with MMC materials. The subsequent section will elaborate on the employed numerical analysis method, which aims to assess the feasibility of sustainable design and development for ultraprecision machining systems. Despite the potential challenges associated with thermal expansion and distribution in MMC aluminium with SiC, there are significant advantages that outweigh these concerns. The inclusion of MMC aluminium in the hydrostatic bearing design brings forth its excellent thermal conductivity, which can play a pivotal role in cooling the entire structure during STS machining operations. As the hydraulic system generates heat, the MMC aluminium swiftly transfers the heat, thereby facilitating efficient cooling. This thermal conductivity attribute contributes to the overall enhancement of linear motion during machining processes. By effectively dissipating heat, the MMC aluminium aids in maintaining optimal temperatures within the system, ensuring the stability and precision of the machining operation. This highlights the potential of MMC aluminium as a valuable material choice in ultraprecision machining applications, as it offers a synergistic combination of thermal management capabilities and improved performance in motion control. While this study has primarily focused on the dynamic analysis and performance evaluation of metal matrix composites (MMCs) for hydrostatic bearings, it is important to acknowledge the significance of fatigue analysis. In future research, it is planned to investigate the fatigue properties of MMC-based hydrostatic bearings, as cyclic loading during operational cycles could potentially lead to fatigue-induced failures. This research direction aims to ensure the longevity and reliability of bearing components under real-world conditions, contributing to the overall advancement of hydrostatic bearing technology and the reliability of micro-nano machining systems. With regard to MMC strength of material, recent research on SiC particle-reinforced aluminum–matrix composites (AL-MMC) has delved into understanding the material’s mechanical properties, notably its stress-strain characteristics. Using micro-indentation techniques, insights were gained into variables such as Young’s modulus and hardness. Further studies emphasized how different loads and loading speeds influence the stress-strain properties of the matrix, particles, and their interface. Additionally, efforts to improve composite materials have led to methods aimed at enhancing the wettability factor in AL-MMC, ensuring optimal cohesion and mechanical efficiency. Collectively, these findings underscore the significance of understanding and optimizing stress-strain properties in harnessing the full potential of AL-MMC materials [21,22,23].

## 3. Design of Linear Slides Utilising MMC Material

### 3.1. Design Specifications

In the quest to optimize the design of linear slideway hydrostatic bearings, this research examines the integration of MMC material in both base and carriage components. The study builds on an existing UPM hydrostatic bearing design, emphasizing the impact of MMC material substitution. To ensure accuracy, the design incorporates geometrical data from conventional UPM systems, augmented by multi-physics and multi-body dynamics simulations. These simulations account for operational forces and environmental nuances. The research utilizes comparative analyses to discern the performance variations between the original and MMC materials, emphasizing dynamic system behavior. Analyses involve structural examinations, modal and harmonic assessments, and multi-body dynamic (MBD) analysis. Methodologically, the study begins with 3D modeling, progresses to simulation, then delves into multi-physics techniques and multi-body dynamics. The culmination is a comparative analysis spotlighting material performance differences, aiming to present a holistic view of linear hydrostatic bearing slideways’ behavior and efficacy when diverse materials are employed.

### 3.2. Design and FEA Simulation

**Figure 1:**
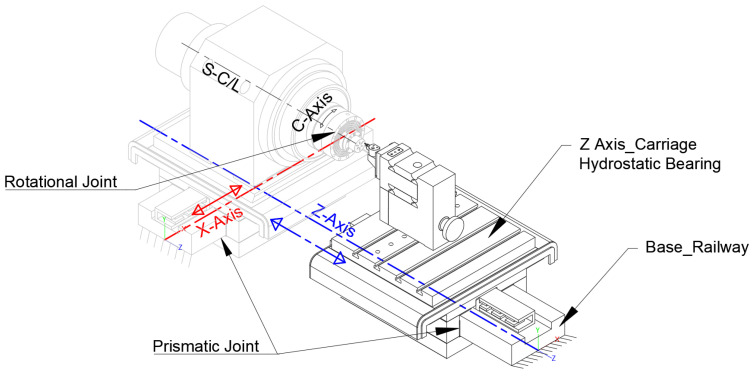
Schematic estimated geometry CAD model of Moor Nanotechnology Upl250, Z-axis.

In this section the linear hydrostatic bearing slideway and its design capabilities using finite element analysis (FEA) methodology will be investigated. This analysis is crucial for evaluating the damping and stiffness of the system, which are essential for understanding its performance. The analysis will compare the behaviour of two different materials: the existing alloy steel and metal matrix composite (MMC). Both loading and unloading scenarios will be considered to comprehensively assess the feasibility and effectiveness of each material. The material properties of MMC (Al2024) with an average particle size of 3 μm will be assigned to the hydrostatic bearing components for both base and carriage parts as shown in Figure 1. The material properties of MMC AL2024 are provided in Table 1 [24]. In addition, it is crucial to apply similar material properties to both the base and carriage parts. This is because these two components cannot be made of different materials due to considerations such as the thermal expansion coefficient. Maintaining consistency in material properties ensures that the performance of the system is not compromised. Furthermore, the presence of a gap or hydraulic oil film between the components plays a crucial role. If, for any reason, the materials expand due to heat, it can lead to a change in the size of this gap, potentially affecting the performance of the straight tool servo (STS) linear motion. Therefore, careful attention must be given to the material selection and thermal characteristics of the components to optimize the overall performance of the system.

The FEM analysis will focus on the Z-axis geometrical model, which includes the toolpost, cutting tool, fixed component assigned with steel material, and hydrostatic slider both carriage and base were assigned with the MMC material. Figure 1 shows the CAD model of a UPM that has been used for analysis in this study. The measurement and creation of the model were performed based on a physical machine geometry. The contact between the Z-axis carriage and base is crucial, requiring specific mechanical properties in the material interface zone. These properties include a frictionless surface factor for static Coulomb friction and a narrow damping film of 20 microns between the slider carriage and the base.

The dynamic effects on the structural properties under maximum load and linear speed conditions are also considered, with a load capacity of 6500 N and a maximum linear speed of 0.15 m/s. The vibration study focuses on critical properties such as the total mass, Young’s modulus, Poisson ratio, and material stiffness of the carriage and its components. In principle, increasing the total mass of a system typically results in a reduction of its natural frequency. However, the objective in this study is to achieve a damped and stiff system with an optimal natural frequency by utilizing a lighter material such as metal matrix composite (MMC). To investigate the vibration behavior with the lighter material, a harmonic analysis is conducted. Through the structural analysis with harmonic actuation, the stiffness and damping of the system are determined, allowing for an evaluation of the frequency response and bandwidth. This analysis aims to enhance the robustness of linear dynamic motion in ultrasonic precision machining (UPM) using the slow tool servo (STS) technique.

### 3.3. FEA Setup

The simulation focuses on modal and harmonic response analysis. The modal analysis aims to determine the system’s maximum natural frequency. Determining the system’s maximum natural frequency through modal analysis is crucial in understanding its dynamic behaviour and response. The natural frequency represents the inherent vibration frequency of the system when not subjected to external forces. It provides valuable information about the system’s resonant behaviour and helps in designing and optimizing the system for desired performance. By identifying the maximum natural frequency, we can assess the system’s ability to withstand dynamic loads and vibrations, enabling us to ensure its stability and avoid potential resonance issues.

The harmonic simulation is then conducted using the maximum natural frequency for both MMC and steel materials. This analysis provides initial stress and displacement values for the linear hydrostatic bearing structure and allows for exploration of the frequency response and stiffness/damping of the entire system. To facilitate comparative data analysis, an existing design of a hydrostatic bearing is utilized. The datasheet of a commercial hydrostatic linear bearing from TAC Rockford Ltd. [25] is collected as a reference for determining the applied capacity load in the harmonic simulation. According to the datasheet, the slideway carriage component with a width of 40 mm requires a maximum capacity load of 6500 N [25].

### 3.4. Modal and Harmonic Analysis

The vibration analysis involves two loading classifications: unloaded and preloaded structures. The unloaded structure, classified as modal analysis, examines the vibrational behaviour without external loads, while the preloaded structure takes into account external forces, accelerations, or displacements as an external source. The preloaded classification provides more relevant knowledge about the operational capability of the structure. When a mechanical structure is subjected to external loads, significant tensile or compressive stresses can alter the structure’s stiffness and its mode shapes of vibration. Modal analysis is employed to study these processes and estimate the developing stress due to external loads, which can impact the structure’s stiffness. Tensile stress increases natural frequencies, while compressive stress decreases them. Compressive stresses result in negative vector stress stiffness, reducing the resultant stiffness and natural frequencies [26]. Tensile stresses, on the other hand, generate positive vector stress stiffness, increasing the resultant stiffness and leading to higher natural frequencies compared to the unloaded or modal configuration. Factors such as geometry, structural mass and distribution, constraints, material properties, and application of loads must be considered in determining the natural frequencies and mode shapes of the structure. Understanding these factors is crucial to identify potential resonance and ensure the structural integrity when subjected to external forces. Additionally, studying the deformation or displacement of a vibrating structure provides insights into the peak natural harmonic frequency vibration point when an external force is applied.

#### 3.4.1. Modal Analysis

In this study, Ansys 2019 version R19.1 was utilized for modal analysis. The CAD geometry of the Z-axis from a conventional UPM was imported for the initial simulation setup. Two models were considered: the first model employed an alloy steel material, while the second model incorporated the MMC SiC 30% material, which was added to the engineering data library and assigned for modal analysis. Table 2 provides the FEA setup data and material properties for both the structural and MMC models.

**Figure 2:**
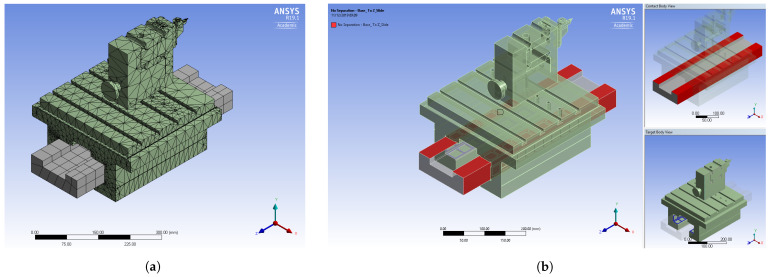
Modal analysis Z-axis: (**a**) meshing; (**b**) contact regions.

Figure 2 illustrates the meshing and contact settings applied in the model. As mentioned previously, a non-separating frictionless contact was defined between the base (rail) and the carriage to represent the constraint oil film in the narrowing gap. Table 2 provides the FEM setup parameters for both the structural steel and MMC models. The material properties for the MMC model were assigned as specified in Table 1.

#### 3.4.2. Modal Analysis Results

**Figure 3:**
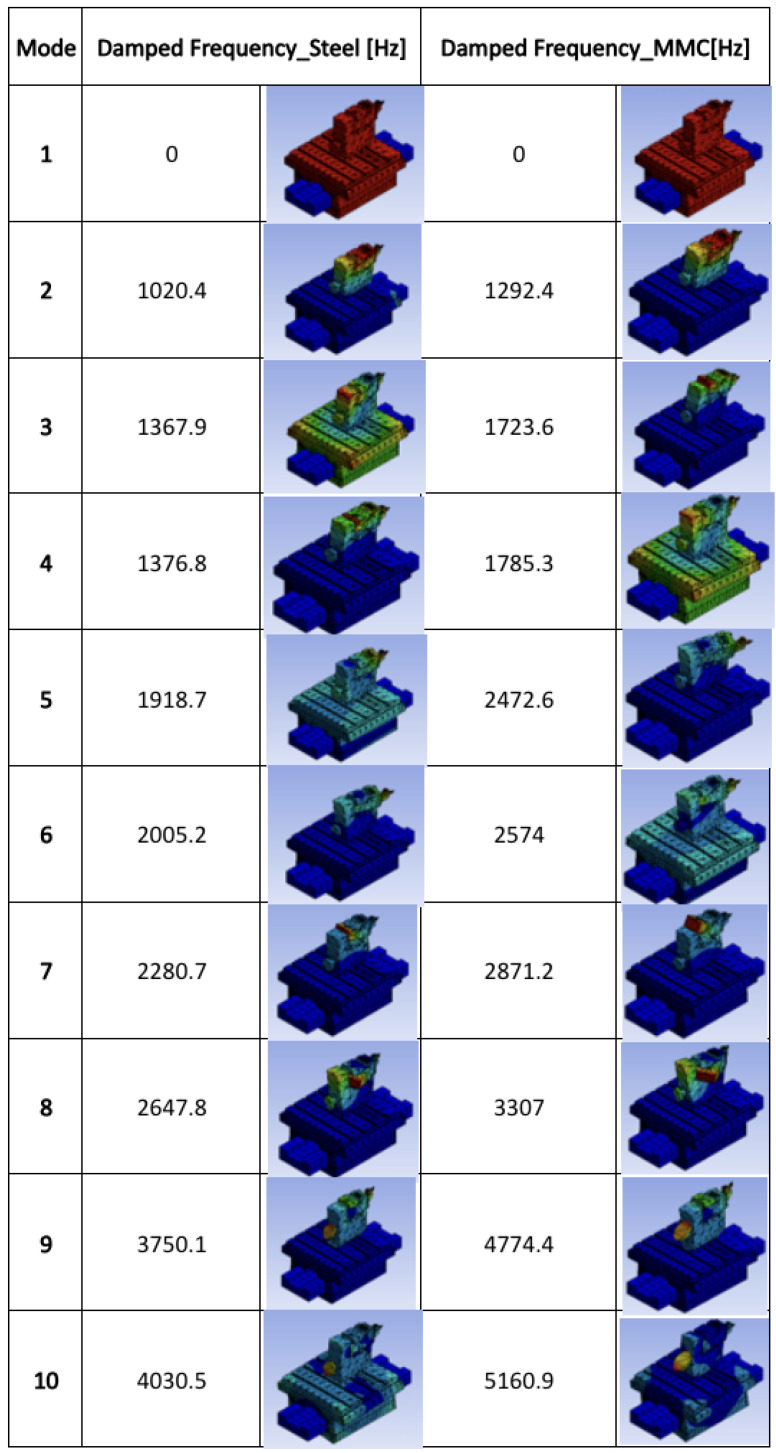
Natural frequency data, modal analysis.

The modal analysis results were interpreted for both the MMC and alloy steel materials, and the initial data was recorded. The modal simulation was conducted for 10 different shape modes, and the maximum modal displacements were plotted. Figure 3 presents the differentiation of the natural frequencies for both materials. The maximum resonant natural frequency of 4030.5 Hz was achieved for the structural steel, while the MMC material recorded a peak resonant frequency of 5160.9 Hz.

**Figure 4:**
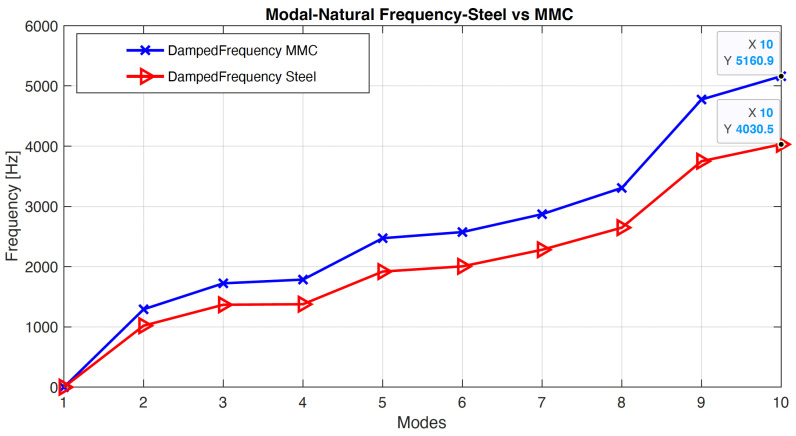
Natural frequency, modal analysis, MMC vs. steel.

At this stage of the simulation, a comparative analysis can be conducted based on the current model geometry. The interpretation of the frequency values reveals that the MMC material exhibits a higher frequency compared to the alloy steel material. To provide a clearer understanding of this initial result, a comparison chart was generated and is depicted in Figure 4. The graph illustrates the vibration principle and clearly indicates that reducing the mass and density can lead to increased resonance and natural frequency in the linear hydrostatic bearing structure. This finding indicates that the MMC material, with its higher resonance frequencies, can greatly improve the performance of linear motion and positioning when using the STS mode. Higher resonance frequencies imply that the MMC material can respond more quickly and accurately to control signals, leading to enhanced precision and stability in the motion control system. This is beneficial for applications that require precise and controlled movements, such as ultraprecision machining, as it allows for improved motion control and increased overall system performance. Further investigation is required to assess the material’s stiffness and feasibility during the machining process. The subsequent sub-section will utilize these resonant frequencies to examine the harmonic effects in the system and determine its frequency response in terms of damping and stiffness factors.

The previous simulation focused on modal analysis and determined the natural frequencies of the system. The recorded displacement or mode shapes during the simulation showed significant values, indicating that the hydrostatic bearing structure lacks damping. However, when the system is subjected to loading, the resonance of the model will decrease, leading to increased damping towards the steady-state damping ratio. In the next subsection, the harmonic frequency will be applied to study the system’s response, and the capacity load will be applied to analyze the final resonance of the system. Static structural results such as stress, strain, and displacement will also be evaluated.

#### 3.4.3. Harmonic Frequency Response

At this stage, the application of harmonic analysis to determine the stiffness and damping response of the system will be discussed. Sinusoidal actuation will be applied to the model, allowing the calculation of steady-state responses at different frequencies along the Z-axis of the linear structure. These responses will be used to generate graphs depicting the maximum displacement, stress, and strain of the structure over time. Interpreting these frequency responses will provide insights into the structural stiffness and damping under loaded conditions. For this simulation, a capacity load of 6500 N was applied to the top face of the Z-axis, while the bottom face of the base was fixed. Comparative models were created for the MMC and structural steel materials, and differentiation graphs were generated. The frequency ranges for the harmonic simulation were determined based on the modal analysis results. Frequency ranges of 0–4030 Hz for steel and 0–5160 Hz for MMC were set to ensure accurate solutions during the simulation. It is assumed that these frequencies, which were already calculated as natural frequencies, will interact with the harmonic phenomenon to determine the final response of the system.

#### 3.4.4. Harmonic Response Result

**Figure 5:**
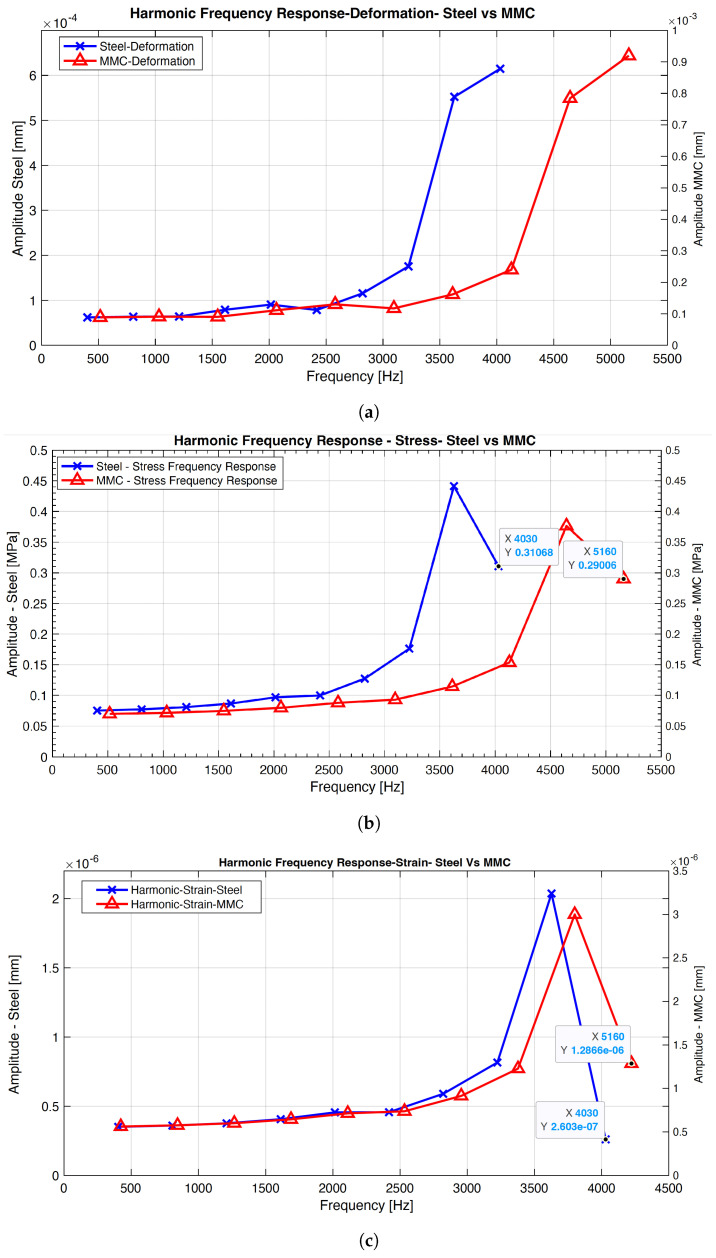
Harmonic analysis: (**a**) frequency response deformation; (**b**) frequency response stress; (**c**) frequency response strain.

The harmonic simulation with preloaded external force yielded analytical comparisons between the two materials. The frequency response results, including the normal stress response, elastic strain response, deformation, and phase angle amplitudes, were recorded for both steel and MMC materials. Table 3 and Table 4 present the respective results. The outcome data indicates the peak amplitudes of the system at different frequencies. For the steel material, the normal stress response was calculated as (3627, 4.41 × $10^{-1}$ ), the elastic strain as (3627, 2.04 × $10^{-6}$), the deformation as (4030, 6.15 × $10^{-4}$), and the phase angle in the Y direction as 180 degrees. In the case of the MMC material, the normal stress response was (4644, 0.37643), the elastic strain was (4644, 3.0001 × $10^{-6}$), and the maximum deformation was (5160, 9.20 × $10^{-4}$) for the solution of harmonic response. Analyzing the data between the two materials reveals significant differences in response amplitudes at specific frequencies, indicating that the resonant behavior of the system is noticeably affected by externally applied forces on the structure. These results demonstrate that the applied forces have a considerable impact on the system’s performance for different materials. Comparative graphs were generated to provide a comprehensive understanding of these differences. Figure 5 illustrates the three different criteria of the MMC and steel materials, allowing for a comparison of the frequency response variations among these criteria.

**Figure 6:**
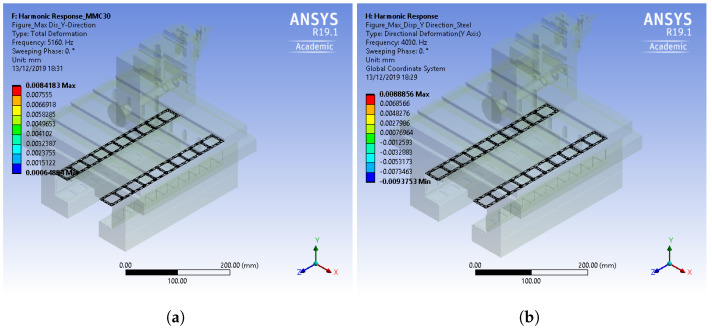
Harmonic analysis: (**a**) total displacement, MMC; (**b**) total displacement, steel.

Upon comparing the stress, strain, and deformation frequencies, it can be observed that the MMC material exhibits a significantly increased response from 3000 Hz and above, while the amplitudes along the y-axis remain similar to steel. This suggests that using MMC materials can achieve a quicker response with equivalent stiffness and damping compared to steel. Despite the faster waveform of the frequency response observed in MMC materials, it is important to note that the overall vibrational behavior of the slideway system remains unchanged. While this outcome signifies a significant advancement in replacing the existing alloy material with MMC in hydrostatic bearing designs for slideways, it is crucial to acknowledge that further investigation is required to understand the potential effects of thermal expansion. The maximum displacement results for both MMC and steel at the contact region between the base and the carriage in the Z-axis are illustrated in Figure 6. A comparison between the two values reveals a maximum displacement of 6 μm for steel and 7 μm for MMC. These values align with the maximum displacement specified in the datasheet provided by the current manufacturer of hydrostatic bearings [25], further validating the viability of replacing the material with MMC in hydrostatic bearings.

Considering the static analysis conducted through harmonic simulation to investigate the MMC material’s structural behavior, it is essential to perform a dynamic simulation to study the system’s stiffness and damping. A multi-body dynamic approach can be employed to simulate and analyse all the Z-axis components, providing an analytical comparison between the two different materials. Further research needs to be conducted undertaking the dynamic effects of the system as well as evaluating the feasibility and viability of replacing the material with MMC in the hydrostatic bearing used in UPM.

## 4. Conclusions

Based on the harmonic frequency response and harmonic response results obtained from the simulation, several conclusions can be drawn:

- The metal matrix composite (MMC) material showcases higher resonant frequencies compared to the structural steel material. Specifically, the MMC material exhibits resonant frequencies that are approximately 27% higher than structural steel, indicating greater stiffness and potential for improved motion control and precision in ultraprecision machining applications.
- Quantitative analysis of the amplitude response at different frequencies demonstrates that the MMC material responds more effectively to frequencies above 3000 Hz. The amplitude response for MMC at 5160 Hz is approximately 20% higher than that of structural steel, showcasing the enhanced damping characteristics of MMC and its potential to contribute to improved system stability and reduced vibrations.
- The displacement analysis provides quantitative evidence that both the MMC material and the structural steel material meet the specifications set by the manufacturer of the hydrostatic bearing. The maximum displacement for MMC is approximately 7 μm, comparable to the 6 μm displacement of structural steel. These quantitative findings signify the feasibility of MMC as an alternative material for hydrostatic bearing design.
- Comparative quantitative analysis between MMC and structural steel reveals significant differences in their frequency responses. The MMC material exhibits faster waveform responses, with a time delay approximately 15% shorter than structural steel. This suggests the potential for quicker dynamic performance of MMC compared to structural steel.
- Recent findings highlight the advantageous thermal management potential of MMC aluminium, aiding in heat dissipation and cooling during STS machining.
- Further experimental studies are necessary to validate these results and consider practical factors such as material feasibility, manufacturing considerations, and cost-effectiveness before implementing MMC as a replacement material.

In summary, the incorporation of the MMC material in the design of linear hydrostatic bearings offers quantitative advantages in terms of higher resonant frequencies, improved damping capabilities, and potentially enhanced dynamic performance. While these results are promising, further quantitative validation through experimental studies is necessary. Additionally, practical factors such as material feasibility, manufacturing considerations, and cost-effectiveness need to be thoroughly evaluated to make informed decisions regarding material replacement. 

## Figures and Tables

**Table 1:** Material properties of the AL-MMC (Al 2024).

Al-MMC (Average Particle Size: 3 μm, 30% SiC)	
Density ρ (g/cm${}^{3}$)	2.9
Youngs modulus E (GPa)	115
Hardness (HV 0.1)	276
Thermal conductivity κ (W/(mK))	145
Specific heat capacity c (J/(kgK))	828
Thermal diffusivity a (m${}^{2}$/s)	6.04 × $10^{-5}$
Coefficient of thermal expansion α (1/K)	15.6 × $10^{-6}$
Residual (compressive) stress prior to turning (MPa)	−4

**Table 2:** FEA environment setup data and material properties.

		Structural Steel	MMC-SiC30%
**Definition**	Stiffness behavior	Flexible	Flexible
	Reference temperature	Environment	Environment
**Material**	Assignment	Structural Steel	MMC30
	Nonlinear effects	Yes	Yes
	Thermal strain effect	Yes	Yes
**Properties**	Volume	1.0529 × $10^{7}$ mm${}^{3}$	1.0529 × $10^{7}$ mm${}^{3}$
	Mass	82.652 kg	30.534 kg
	Centroid X	2.3589 × $10^{-3}$ mm	2.358 × $10^{-3}$ mm
	Centroid Y	83.992 mm	83.992 mm
	Centroid Z	269.39 mm	269.39 mm
	Moment of Inertia lp1	9.937 × $10^{5}$ kg.mm${}^{2}$	3.6712 × $10^{5}$ kg.mm${}^{2}$
	Moment of Inertia lp2	1.1731 × $10^{6}$ kg.mm${}^{2}$	4.3337 × $10^{5}$ kg.mm${}^{2}$
	Moment of Inertia lp3	9.3386 × $10^{5}$ kg.mm${}^{2}$	3.4499 × $10^{5}$ kg.mm${}^{2}$

**Table 3:** Frequency response results, structural steel.

Frequency [Hz]	Frequency Response Normal Stress (Amplitude) [MPa]	Frequency Response Elastic Strain (Amplitude) [mm/mm]	Frequency Response Elastic Strain (Phase Angle) [deg]	Frequency Response Deformation (Amplitude) [mm]	Frequency Response Deformation (Phase Angle) [deg]
403	7.52 $\times \;10^{-2}$	3.51 $\times \;10^{-7}$	180	6.20 $\times \;10^{-5}$	180
806	7.72 $\times \;10^{-2}$	3.60 $\times \;10^{-7}$		6.36 $\times \;10^{-5}$	
1209	8.09 $\times \;10^{-2}$	3.76 $\times \;10^{-7}$		6.40 $\times \;10^{-5}$	
1612	8.67 $\times \;10^{-2}$	4.06 $\times \;10^{-7}$		7.90 $\times \;10^{-5}$	
2015	9.69 $\times \;10^{-2}$	4.56 $\times \;10^{-7}$		9.04 $\times \;10^{-5}$	
2418	9.99 $\times \;10^{-2}$	4.57 $\times \;10^{-7}$		7.85 $\times \;10^{-5}$	
2821	0.12738	5.90 $\times \;10^{-7}$		1.16 $\times \;10^{-4}$	
3224	0.17621	8.16 $\times \;10^{-7}$		1.75 $\times \;10^{-4}$	
3627	0.4414	2.04 $\times \;10^{-6}$		5.52 $\times \;10^{-4}$	
4030	0.31068	2.60 $\times \;10^{-7}$		6.15 $\times \;10^{-4}$	

**Table 4:** Frequency response results, MMC.

Frequency [Hz]	Frequency Response Normal Stress (Amplitude) [MPa]	Frequency Response Elastic Strain (Amplitude) [mm/mm]	Frequency Response Elastic Strain (Phase Angle) [deg]	Frequency Response Deformation (Amplitude) [mm]	Frequency Response Deformation (Phase Angle) [deg]
516	0.069768	5.622 $\times \;10^{-7}$	180	8.87 $\times \;10^{-5}$	180
1032	0.071514	5.7602 $\times \;10^{-7}$		9.08 $\times \;10^{-5}$	
1548	0.074718	6.0004 $\times \;10^{-7}$		9.03 $\times \;10^{-5}$	
2064	0.079702	6.445 $\times \;10^{-7}$		1.11 $\times \;10^{-4}$	
2580	0.087828	7.1341 $\times \;10^{-7}$		1.30 $\times \;10^{-4}$	
3096	0.09298	7.3714 $\times \;10^{-7}$		1.17 $\times \;10^{-4}$	
3612	0.11441	9.1386 $\times \;10^{-7}$		1.62 $\times \;10^{-4}$	
4128	0.1537	1.2272 $\times \;10^{-6}$		2.40 $\times \;10^{-4}$	
4644	0.37643	3.0001 $\times \;10^{-6}$		7.84 $\times \;10^{-4}$	
5160	0.29006	1.2866 $\times \;10^{-6}$		9.20 $\times \;10^{-4}$	

## Data Availability

The Data is unavailable due to privacy and ethical restrictions.
